# Co-infection with SARS-CoV-2 Omicron and Delta variants revealed by genomic surveillance

**DOI:** 10.1038/s41467-022-30518-x

**Published:** 2022-05-18

**Authors:** Rebecca J. Rockett, Jenny Draper, Mailie Gall, Eby M. Sim, Alicia Arnott, Jessica E. Agius, Jessica Johnson-Mackinnon, Winkie Fong, Elena Martinez, Alexander P. Drew, Clement Lee, Christine Ngo, Marc Ramsperger, Andrew N. Ginn, Qinning Wang, Michael Fennell, Danny Ko, Linda Hueston, Lukas Kairaitis, Edward C. Holmes, Matthew N. O’Sullivan, Sharon C.-A. Chen, Jen Kok, Dominic E. Dwyer, Vitali Sintchenko

**Affiliations:** 1 Sydney Institute for Infectious Diseases, University of Sydney, Sydney, NSW Australia; 2 Centre for Infectious Diseases and Microbiology-Public Health, Westmead Hospital, Westmead, NSW Australia; 3 Institute for Clinical Pathology and Medical Research, New South Wales Health Pathology, Westmead, NSW Australia; 4 Renal Services Blacktown Hospital, Western Sydney Local Health District, Sydney, NSW Australia; 5 School of Life and Environmental Sciences, University of Sydney, Sydney, NSW Australia; 6 School of Medical Sciences, University of Sydney, Sydney, NSW Australia

**Keywords:** Epidemiology, Microbial genetics, Infectious-disease diagnostics

## Abstract

Co-infections with different variants of SARS-CoV-2 are a key precursor to recombination events that are likely to drive SARS-CoV-2 evolution. Rapid identification of such co-infections is required to determine their frequency in the community, particularly in populations at-risk of severe COVID-19, which have already been identified as incubators for punctuated evolutionary events. However, limited data and tools are currently available to detect and characterise the SARS-CoV-2 co-infections associated with recognised variants of concern. Here we describe co-infection with the SARS-CoV-2 variants of concern Omicron and Delta in two epidemiologically unrelated adult patients with chronic kidney disease requiring maintenance haemodialysis. Both variants were co-circulating in the community at the time of detection. Genomic surveillance based on amplicon- and probe-based sequencing using short- and long-read technologies identified and quantified subpopulations of Delta and Omicron viruses in respiratory samples. These findings highlight the importance of integrated genomic surveillance in vulnerable populations and provide diagnostic pathways to recognise SARS-CoV-2 co-infection using genomic data.

## Abstract (web summary)

Here, using genomic approaches, Rockett et al. identify Omicron and Delta SARS-CoV-2 co-infections in two adults, highlighting the usefulness of genomic surveillance for the timely recognition of co-infections in situations when different variants of the virus are circulating in the community.

## Introduction

Since the declaration of the COVID-19 pandemic by the World Health Organization on March 11th 2020, SARS-CoV-2 has gradually evolved into phylogenetically distinct lineages, some of which have been designated variants of concern (VOCs)^1,2^. These variants differ in terms of transmissibility, capacity to cause severe disease and the ability to evade post-vaccination derived immunity. The prevalence of individual VOCs in different global regions has been affected by the timing and location of their emergence and the corresponding measures for COVID-19 control^3^. Development and implementation of viral genomic surveillance and rapid sharing of genomic data has provided a critical capacity to distinguish and monitor SARS-CoV-2 variants and conduct risk assessments of their significance. Co-infection with different SARS-CoV-2 lineages was rarely reported during the first COVID-19 wave in 2020 prior to the introduction of vaccination programmes^4–6^, but it has been suggested that such co-infections could lead to greater severity and disease duration^4^. However, co-infections involving either VOC Delta or VOC Omicron have not yet been reported, nor have they been documented in immunosuppressed hosts, which may drive saltational evolution characterised by high numbers of new mutations^7^. As reports of SARS-CoV-2 recombinants emerge, understanding the frequency and drivers of recombination events that occur during SARS-CoV-2 co-infection becomes essential^8^. In this study we report cases of co-infection with Delta and Omicron in two immunocompromised individuals at risk of severe COVID-19 disease identified during local co-circulation of both SARS-CoV-2 lineages.

## Results

### Clinical and epidemiological context

Case A was a patient who returned a positive SARS-CoV-2-specific polymerase chain reaction (PCR) result from a nasopharyngeal swab after presenting to the Emergency Department of a Sydney hospital with mild respiratory symptoms. Case B was a patient diagnosed by SARS-CoV-2 PCR after presenting to the same hospital with fever. Further analysis of health records revealed that both patients had chronic kidney disease due to type 2 diabetes, obesity and ischaemic heart disease. In addition, both were receiving haemodialysis treatment for 4–5 h thrice weekly at the same community dialysis centre and were therefore potentially exposed to multiple and infectious COVID-19 cases during treatment sessions. Given the high community incidence of COVID-19, infection control measures implemented at the dialysis centre to prevent nosocomial transmission included physical distancing and masking of patients at all times, decontamination of treatment stations and dialysis equipment after each session, four-point personal protective equipment use by all clinical staff and regular testing of patients by SARS-CoV-2 PCR at the time of each treatment. Despite the similarities in patient demographics, they were unknown to each other, had not received haemodialysis at the same time nor used the same equipment or treatment station.

### SARS-CoV-2 viral load and testing for other respiratory viruses

Two respiratory swabs collected from Case A on days 2 and 3, as well as two respiratory swabs from Case B collected on days 3 and 11, were subjected to nucleic acid extraction, quantitative SARS-CoV-2 PCR. Viral yield in samples was variable but still significant and suggesting the presence of viable virus (Table 1). PCR did not detect human influenza viruses A or B, respiratory syncytial virus, parainfluenza viruses 1, 2, and 3, human metapneumovirus or rhinovirus in samples from either case.

**Table 1:** SARS-CoV-2 yield in Cases A and B.

Samples	SARS-CoV-2 PCR Ct value	SARS-CoV-2 viral load		PANGO Lineage
		Copy/µl	Log_10_	
Case A				
Day 0^a^	28.18	5713	3.8	Omicron BA.1; Delta AY.39.1
Day 2	17.33	98,130,334	8.0	Omicron BA.1; Delta AY.39.1
Day 3	23.44	404,527	5.6	Omicron BA.1; Delta AY.39.1
Day 3 culture	15.37	571,255,772	8.8	Delta AY.39.1
Case B				
Day 0	31.68	246	2.4	Omicron BA.1
Day 3	19.26	17,317,514	7.2	Omicron BA.1; Delta AY.39.1
Day 11	24.05	233,804	5.4	Omicron B.1.1.529; Delta AY.39.1

^a^Specimens only sequenced using Illumina methodology due to low viral load.

### COVID-19 serology and vaccination history

Neither patient had prior evidence of COVID-19 infection. A previously described immunofluorescence assay (IFA)^9^ performed on sera collected from Case A on day 3 and Case B 5 months prior to the diagnosis did not detect SARS-CoV-2 antibodies (i.e. IgG, IgA and IgM IFA titres all <10, trimeric spike IgG negative, nucleoprotein IgG negative). Case B had received two doses of the COMIRNATY® (Pfizer) vaccine with the second dose 10 weeks prior to diagnosis. Case A remained unvaccinated by choice.

### SARS-CoV-2 viral culture

Viral culture of the Case A, day 3 sample yielded Delta 4 days post-infection, the consensus genome recovered from this culture matched the genome reconstructed from the mixed sample. It is likely that Delta had overgrown Omicron as TMPRSS2 enhanced VeroE6 cells are less permissible to Omicron, but highly adapted to Delta infection^10^. Viral culture was retrospectively and unsuccessfully attempted for the specimen collected from Case A, day 2.

### SARS-CoV-2 genomic analysis

Samples from both cases (Case A—day 2 after onset, Case B—day 3) underwent whole genome sequencing as part of the prospective genomic surveillance programme in New South Wales, Australia^11^. A sample from an epidemiologically linked household contact of Case A, Case C, who was diagnosed several days after Case A was also sequenced.

**Fig. 1:**
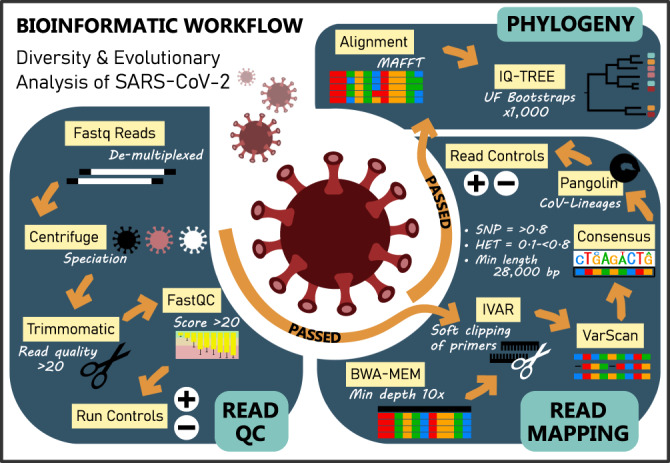
Overview of SARS-CoV-2-specific bioinformatic workflow. Created using Affinity Designer v.1.10.5.1342.

In our public health surveillance system only genomes confidently assigned to SARS-CoV-2 lineages (see Fig. 1 for quality framework) are reported to the health authorities and shared globally via GISAID (https://www.gisaid.org). In contrast to the majority of community samples sequenced, a small proportion had unexpectedly high numbers of “heterozygous” (i.e., mixed nucleotides at a single site) calls (Supplementary Fig. 1) and could not be unambiguously assigned to a SARS-CoV-2 lineage by the Pangolin software. This observation triggered a case review in January 2022 to identify cases for which these results could be confirmed by testing of additional samples. Two cases (i.e. Cases A and B) with additional respiratory samples available for genome sequencing were selected.

**Fig. 2:**
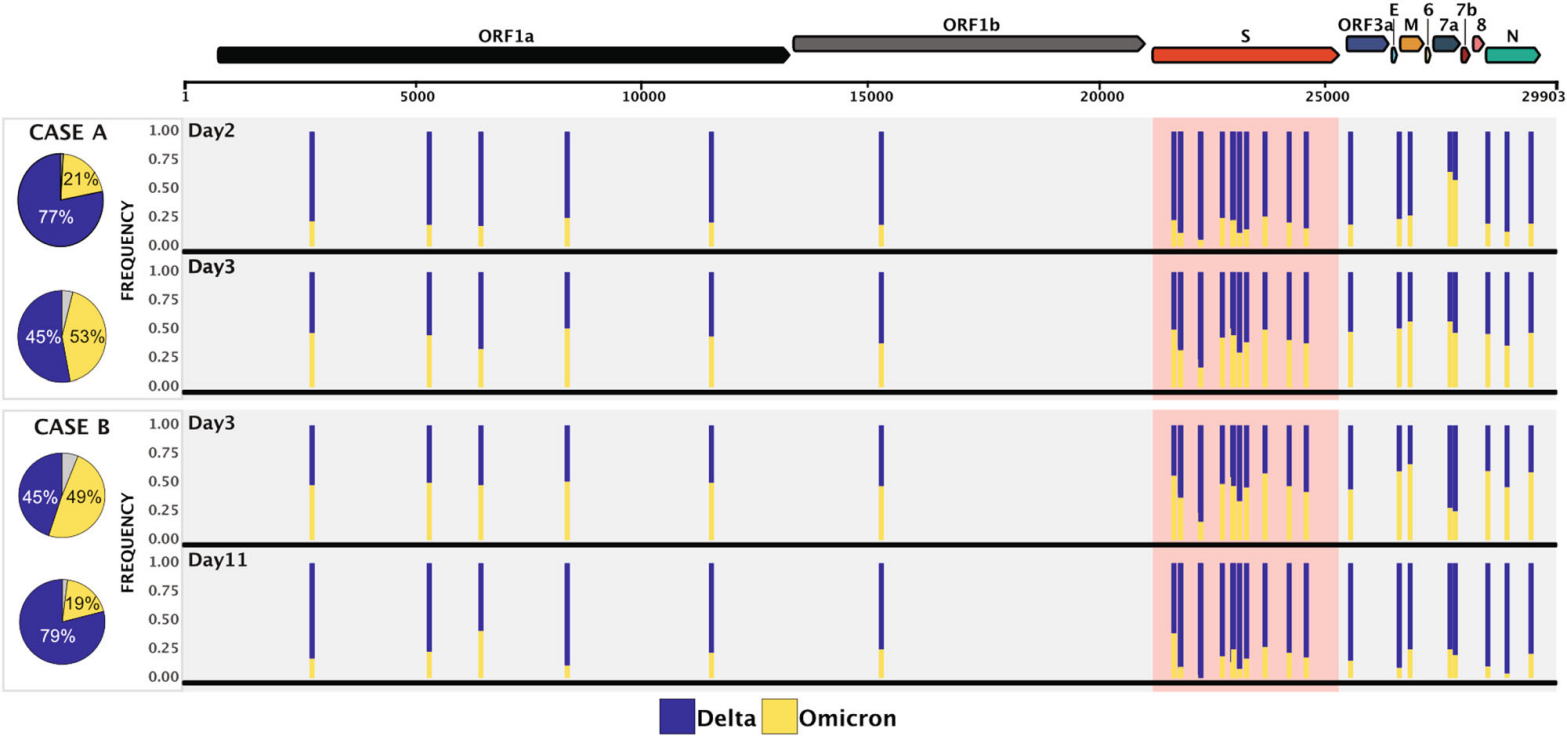
Genome-wide view of the variant frequency of the SARS-CoV-2 Delta and Omicron lineage-defining polymorphisms in specimens sequenced using the RVOP SARS-CoV-2 enrichment protocol. Pie graphs depict the average population frequency of Omicron and Delta lineage-defining mutations in four clinical samples collected from Cases A and B. Segments in grey represent differences between the average read frequency of Omicron and Delta markers. A total of 27 polymorphisms defining Delta and Omicron lineages are presented in relation to the annotated SARS-CoV-2 genome. The frequency of sequencing reads encoding each mutation is shown by histograms highlighting the constellation of mutations defining each lineage. Blue bars demonstrate the frequency of mutations defining the Delta lineage and yellow bars the Omicron lineage. Due to the close genomic location of lineage-defining mutations in the spike region some bars are overlapping. Read frequencies shown were collected from RVOP data but were highly concordant between sequencing methods and technologies.

**Fig. 3:**
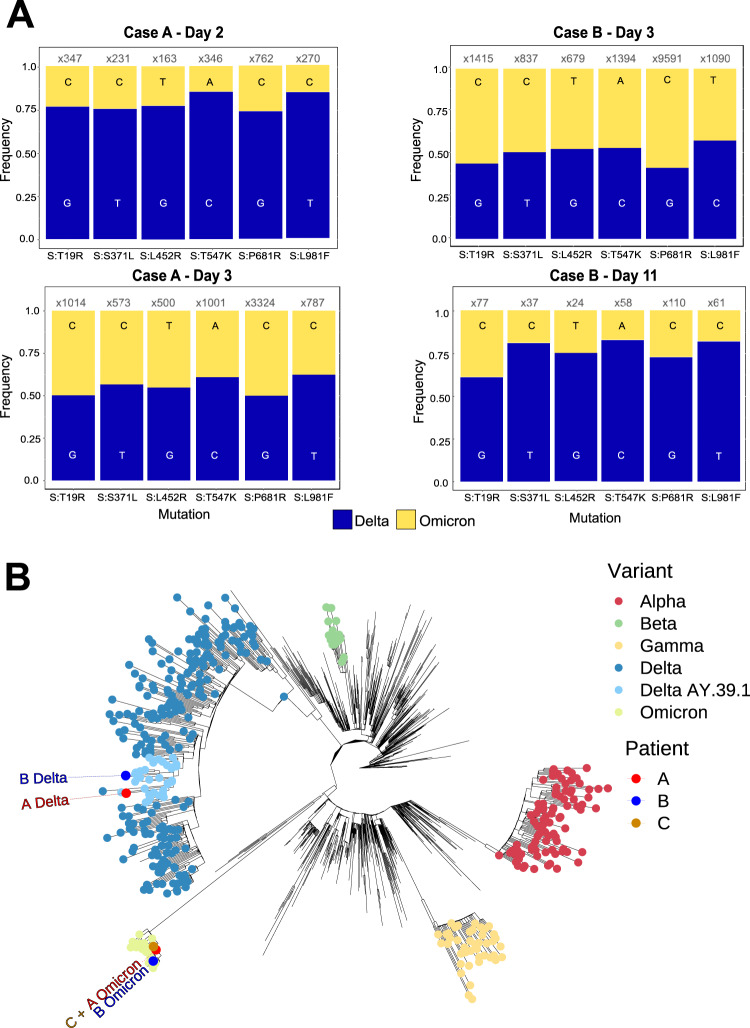
Population and phylogenetic analysis of two cases of SARS-CoV-2 co-infection with Delta and Omicron VOCs. **A** Population analysis of key lineage-defining mutations in the SARS-CoV-2 spike gene for each specimen. Nucleotide frequency and relative coverage of genomic regions specific for either Omicron or Delta. The *X*-axis represents genomic positions and *Y*-axis indicates their relative frequencies derived from RVOP data. **B** Unrooted maximum likelihood phylogeny representing the sequences obtained from Cases A, B and C in the context of global diversity of SARS-CoV-2. Genomes generated as part of this study are labelled individually. The predominant Delta lineage in Australia, AY.39.1, is highlighted. The Delta strains from cases A and B are from separate clades of AY.39.1 circulating in Australia, whereas the two Omicron strains are both in the same sub-lineage of Omicron (BA.1) which dominated in Australia in December 2021–January 2022. Note that the Omicron samples from patients A and C are identical and hence overlap. Branch lengths are scaled according to the number of nucleotide substitutions per site.

Due to the low viral load in the day 0 samples from both cases, they were only able to be sequenced using Midnight primers and Illumina sequencing, while two longitudinal samples from each case with increased viral loads were subjected to further analyses (Supplementary Table 1). Genome sequencing was achieved using short-read (NextSeq 500 (Illumina)) and long-read (GridION (Oxford Nanopore Technologies; ONT)) protocols with Midnight primers. We also employed the probe-based Illumina Respiratory Viral Oligo panel (RVOP)^12^ to collect reads unbiased by SARS-CoV-2 PCR amplification. Careful examination of the relative frequency of 17 Omicron lineage-defining markers and 10 Delta lineage-defining markers^13^ clearly demonstrated co-infection with both lineages (Figs. 2 and 3A, Supplementary Table 1, Supplementary Data 1–3, Supplementary Dataset). The overall proportions of Delta and Omicron were highly concordant between all three sequencing methods (Supplementary Fig. 2), however four lineage markers showed evidence of amplification bias when SARS-CoV-2 was amplified using Midnight primers (Supplementary Fig. 4). Population analysis of genomic data generated using RVOP methods estimated that the VOC proportions in samples from Case A were 21% Omicron and 77% Delta on day 2, compared to 45% Omicron and 53% Delta on day 3. Samples from Case B contained 42% Omicron and 53% Delta on day 3, as well as 11% Omicron and 84% Delta on day 11 (Fig. 2 and Supplementary Figs. 2–4). Despite the similar pattern of mixed infection, the two cases were not genomically linked (Fig. 3B). The two Omicron sequences were distinct representatives of the Omicron (lineage B.1.1.529, sub-lineage BA.1) strain predominating in Sydney at the time, while the two Delta sequences belonged to different genomic clusters of Delta (lineage B.1.617.2, sub-lineage AY.39.1) also circulating locally (Fig. 3 and Table 1). These conclusions were supported by matching the Omicron sequence from Case A to the Omicron sequence recovered from their household contact, Case C (Fig. 3). No recombination events were observed (Supplementary Data 3).

## Discussion

Our findings demonstrated two independent episodes of SARS-CoV-2 co-infection with VOCs Delta and Omicron in epidemiologically unrelated adult patients requiring maintenance haemodialysis. Although we detected phylogenetically distinct SARS-CoV-2 variants in both cases, our results cannot differentiate if cases acquired their dual SARS-CoV-2 co-infection following sequential exposures to individuals with a single lineage infection. Further studies of SARS-CoV-2 within-host population dynamics are required to better understand these processes. The most likely hypothesis for these two cases of SARS-CoV-2 co-infection is the sustained exposure of susceptible, immunosuppressed hosts to multiple patients infected with Delta or Omicron at a time when there was widespread community circulation of both VOCs. Case B appeared to be initially infected by Omicron and subsequently superinfected with Delta shortly prior to hospital admission as there were only Omicron sequences in the day 0 sample and high viral loads of Omicron and Delta obtained from the day 3 sample. SARS-CoV-2 infections in haemodialysis patients, including vaccine breakthrough infections, are often nosocomial and present significant mortality risks^14^. Comparing the outcomes of these two patients to reference populations is difficult as severity of presentations can be influenced by multiple factors, including comorbidities, the viral variant and vaccination status.

The identification of phylogenetically distinct and epidemiologically relevant SARS-CoV-2 variants within the same host further expands the significance of genomic surveillance and highlights the added value of patient and public health context during clinical genomics analysis. The timely recognition of mixed infections may also affect the selection of appropriate antiviral therapy and infection control measures. Whilst the multiple sequencing methods presented here demonstrated concordant results (Supplementary Figs. 2 and 4), not all of them are required to investigate every suspected co-infection case. The RVOP approach was particularly informative as it captured several lineage markers affected by amplification bias due to polymorphisms in whole genome sequencing primers (Supplementary Material, Supplementary Figs. 2–4). We acknowledge that VOC Omicron was responsible for about 68% of SARS-CoV-2 genomes sequenced in the state during the study period, and that Cases A and B had relatively equal proportions of sequences representing two lineages in at least one timepoint. The confident identification of minority populations of lineage-specific sequences (e.g., <10%) in more unbalanced populations might be significantly harder; access to longitudinal samples with sufficient viral load is needed to address these challenges.

Genomic epidemiology has rapidly become a high-resolution tool for local and international public health surveillance and disease control. However, the international coverage of SARS-CoV-2 genomic surveillance remains heavily biased towards countries with specialised genomic facilities and research programmes^3,15^. Furthermore, genomic surveillance relies on data sharing by multiple and geographically distributed providers which employ different sequencing and bioinformatic techniques. The reliance on consensus genome data and strict data quality criteria used by genomic laboratories and data sharing environments were designed to minimise the noise from laboratory contamination events and sequencing imperfections. However, such quality metrics can, by design, filter out potentially significant cases associated with high heterozygosity due to mixed viral populations as presented here.

In conclusion, these findings demonstrated the capacity of clinically and epidemiologically informed genomic surveillance to diagnose co-infections with significant SARS-CoV-2 variants and highlight the needed for deeper analysis of genomic surveillance data in clinical and public health contexts. Identifying dual infections may guide the use of monoclonal antibodies that have reduced activity to the VOC Omicron. SARS-CoV-2 co-infections, particularly when they occur in vulnerable hosts, may underpin saltational evolution, thus emphasising the important role COVID-19 genomic surveillance will play in diagnostic virology, in the era of mass vaccination.

## Methods

### Respiratory virus detection by RT-PCR

RNA was extracted using either the Viral NA Small volume kit on the MagNA Pure 96 system (Roche Diagnostics GmbH) or RNeasy mini-kit (Qiagen). Both protocols used 200 μl of clinical specimen, although minor modifications were performed during the RNeasy extraction, as previously described^12^. A final elution volume of 100 μL was used during MagNA Pure 96 system whereas the RNeasy extraction elute was 30 μL.

A previously described RT-PCR, targeting the SARS-CoV-2 nucleocapsid gene was employed to estimate the viral load of clinical specimens from the RNeasy extraction^16^. A commercially available synthetic RNA control (Wuhan-Hu-1 reference sequence, TWIST Biosciences NCBI GenBank accession MN908947.3) was used in ten-fold dilutions starting at 20,000 copies/µL to 2 copies/µL to generate a standard curve and quantify the viral load per microlitre of extracted RNA per specimen (cpy/μL). Additional RT-PCRs were used to investigate the presence of common viral respiratory viruses: human influenza viruses A and B, parainfluenza viruses 1, 2, and 3, respiratory syncytial virus, adenovirus, and rhinovirus^17^.

### SARS-CoV-2 culture

Respiratory specimens were cultured in VeroE6 cells expressing transmembrane serine protease 2 (VeroE6/TMPRSS2; JCRB1819) as previously outlined^18^. Briefly, cell cultures were seeded at 1–3 × 10^4^ cells/cm^2^ in Dulbecco’s minimal essential medium (Lonza, Basel, Switzerland) supplemented with 9% foetal bovine serum (FBS, HyClone). Media was replaced within 12 h with inoculation media containing 1% FBS with the addition of penicillin (10,000 U/mL), streptomycin (10,000 µg/Ll) and amphotericin B deoxycholate (25 µg/mL) (Lonza) to prevent microbial overgrowth and then inoculated with 100 µL of SARS-CoV-2 RT-PCR positive respiratory sample. The inoculated cultures were incubated at 37 °C in 5% CO_2_ for 4 days and observed daily for cytopathic effect (CPE). Routine mycoplasma testing using RT-PCR was performed to exclude cell line mycoplasma contamination and culture work was undertaken under physical containment laboratory level 3 (PC3) biosafety conditions. The presence of CPE and increasing viral load as measured by the before mentioned SARS-CoV-2 RT-PCR was indicative of positive SARS-CoV-2 culture. RNA extracts were also subjected to the SARS-CoV-2 genomics workflow as described below.

### Frequency of SARS-CoV-2 variant heterozygosity

The frequency of “heterozygosity” (i.e., mixed nucleotides at a single site) during SARS-CoV-2 variant calling is monitored as part of our in-house bioinformatic quality control system, as is the inability to determine a SARS-CoV-2 Pango lineage designation on an otherwise complete and high coverage genome (Supplementary Fig. 1). These two markers signal that a specimen requires repeat extraction, SARS-CoV-2 amplification, library preparation and sequencing.

### SARS-CoV-2 whole genome amplification

Tiling PCR was used to amplify the entire SARS-CoV-2 genome from RNA extracts of clinical specimens using primers outlined in the Midnight sequencing protocol^4^. Each PCR included 12.5 µL Q5 High Fidelity 2x Master Mix (New England Biolabs), 1.1 µL of either pool 1 or pool 2 10 µM primer master mix, 2.5 µL of template RNA and molecular grade water was added to generate a total volume of 25 µL. Cycling conditions were: initial denaturation at 95 °C for 2 min, then 35 cycles of: 95 °C for 30 s, 65 °C for 2 min 45 s, and a final extension step of 75 °C for 10 min. Pool 1 and pool 2 amplicons were combined and purified with a 1:1 ratio of AMPureXP beads (Beckman Coulter) and eluted in 30 µL of RNAase free water. Purified products were quantified using Qubit™ 1x dsDNA HS Assay Kit (Thermo Fisher Scientific) and diluted to the desired input concentration for library preparation.

### Amplicon short-read library preparation

Purified amplicon pools were used to generate sequencing libraries using Nextera XT (Illumina) according to the manufacturer’s instructions and pooled with the aim of producing 1 × 10^6^ reads per library. Sequencing libraries were then sequenced with paired-end 76-bp chemistry on the iSeq, MiniSeq or NextSeq (Illumina) instruments.

### Amplicon ONT library preparation

In parallel, the purified amplicon pools were also utilised to generate libraries using SQK-RBK004 (ONT) according to the manufacturer’s instructions, loaded onto a R9.4.1 flow cell. Sequencing was performed on the GridION platform running MinKNOW version 21.05.25 with live base-calling on high accuracy mode with demultiplexing enabled (Guppy version 5.0.16). Sequencing run status was monitored on board MinKNOW and run was terminated after more than 20 MB of passed base-called data was obtained per sample.

### Respiratory viral enrichment using hybridisation capture probes

The cDNA generated prior to whole genome amplification was used as input into the RNA Prep with Enrichment kit (Illumina). Second-strand cDNA synthesis, cDNA tagmentation, library construction, clean-up, and normalisation were performed according to manufacturer’s instructions. Individual libraries were then combined in 3-plex reactions for probe hybridisation. The RVOP v2 (Illumina) was used for probe hybridisation with the final hybridisation step held at 58 °C overnight. Hybridised probes were then captured and washed according to manufacturer’s instructions and amplified as follows: initial denaturation 98 °C for 30 s, 14 cycles of: 98 °C for 10 s, 60 °C for 30 s, 72 °C for 30 s, and a final 72 °C for 5 min. Library quantities and fragment size were determined using a Qubit 1× dsDNA HS assay and Agilent HS Tapestation and sequenced using 2 × 76-bp runs on the iSeq (Illumina).

### Bioinformatic analysis of Illumina data

Raw sequence data files were processed using an in-house quality control procedure prior to further analysis (Fig. 1) as described previously^11,12^. De-multiplexed reads were quality trimmed using Trimmomatic v0.36 (sliding window of 4, minimum read quality score of 20, leading/trailing quality of 5 and minimum length of 36 after trimming)^19^. Briefly, reads were mapped to the reference SARS-CoV-2 genome (NCBI GenBank accession MN908947.3) using Burrows-Wheeler Aligner-mem version 0.7.17^20^, with unmapped reads discarded. Average genome coverage was estimated by determining the number of missing bases (Ns) in each sequenced genome. For amplicon generated reads variant calling and the generation of consensus sequences was conducted using iVar^21^, with soft clipping over primer regions (version 1.2.1, min. read depth >10x, quality >20, min frequency threshold of 0.1). Single nucleotide polymorphisms (SNP) were defined based on an alternative frequency >0.75 whereas minority allele frequency variants (MFV) were defined by an alternative frequency between 0.1 and 0.75. Variants falling in the 5’ and 3’UTR regions were excluded. Polymorphic sites that have previously been highlighted as problematic were monitored^22^. To ensure the accuracy of variant calls only genomes with >90% genome coverage and a mean depth of >100x were included. The MFV calls were excluded in the base pair either side of the 5’ or 3’-end of indels due to potential mis-mapping. SARS-CoV-2 lineages were inferred using Phylogenetic Assignment of Named Global Outbreak LINeages v1.2.86 (PANGO and PLEARN)^23,24^.

### Bioinformatic analysis of ONT data

Quality control and consensus sequences were generated post run using the wf-artic workflow version 0.3.9 (https://github.com/epi2me-labs/wf-artic). To determine and quantify positional heterozygosity, mapping files generated by the wf-artic workflow were visualised on the Integrative Genomics Viewer^25^ version 2.8.6 and parsed using bam-readcount version 1.0.1 (https://github.com/genome/bam-readcount).

### Investigation of amplification bias in co-infection cases

Consensus and MFV were collated over constellations of mutations that define the SARS-CoV-2 lineages B.1.617.2 (Delta) and B.1.1.529, sub-lineage BA.1 (Omicron) for each genome investigated (https://github.com/cov-lineages/constellations/blob/main/constellations/). This included ten unique genomic locations that define Delta (B.1.617.2) (S:T19R, S:L452R, S:P681R, ORF3a:S26L, M:I82T, ORF7a:V82A, ORF7a:T120I, N:D63G, N:R203M, N:D377Y). Mutations that co-exist in BA.1 were not included (S:G142D, S:T478K, S:D950N). In addition to 17 polymorphisms that define the dominant Omicron sub-lineage BA.1 (orf1ab:K856R, del:6513:3, nuc:T5386G, orf1ab:A2710T, orf1ab:I3758V, nuc:C15240T, S:A67V, del:21765:6, del:21987:9, del:22194:3, nuc:22205 + GAGCCAGAA, S:S371L, S:G446S, S:G496S, S:T547K, S:N856K, S:L981F, M:D3G) mutations that co-exist in B.1.617.2 where not included (S:T95I). Alternative read frequencies over each polymorphism were compared using all three sequencing techniques and between sampling timepoint of each case.

### Phylogenetic analysis of reconstructed strains

Representative SARS-CoV-2 genomes collected between December 2020 and 31st December 2022 (*n* = 1300, ≥27,000-bp in length) were downloaded from the Global Initiative on Sharing All Influenza Data (GISAID)^26^ EpiCoV, using a global subsampling strategy developed by Nextstrain^27^. Phylogenetic inference and visualisation was performed using 1081 high quality consensus SARS-CoV-2 FASTA sequences (GISAID, *n* = 1076; this study, *n* = 5). The GISAID data set included representatives of primary Delta and Omicron strains documented as circulating in Sydney at the time of the study. For Case B, the consensus genomes for the day 0 (Omicron only, Illumina Midnight) and day 3 (Delta dominant, Illumina RVOP to avoid Midnight amplification bias) samples were used. The Delta sequence for Case A, for which all three samples were of mixed lineage, was obtained from the day 3 sample viral culture. To obtain the Omicron sequence for Case A, the RVOP Illumina data from the day 3 sample that the culture was derived from was hand-reviewed to generate a consensus containing only the Omicron-specific SNPs, minus the Delta-specific SNPs seen in the culture sequence. The resulting reconstructed Case B Omicron genome was then compared to the Omicron sequence from Case C, who was assumed to have caught their infection from Case B; these two sequences matched. These four sequences, as well as the downloaded sequences from GISAID, were trimmed to remove the 5’ and 3’ UTR regions and aligned with MAFFT v7.402 (FFT-NS-2, progressive method)^28^. Phylogenetic analysis was performed using the maximum likelihood approach (IQTree v1.6.7 (substitution model: GTR + F + R2)) with 1000 bootstrap replicates^29^. The phylogenetic tree was visualised using the R package ggtree version 1.99.1^30^.

### Statistical analysis

Statistical analysis of the read distribution of Delta and Omicron lineage markers was performed by Welch two sample *t*-test using R software version 4.1.2^31^ (2021-11-01) with the t.test function from the package ‘stats’. Graphs were generated using the package ‘ggplot 2’ version 3.3.5^32^.

### Human research ethics approval

Human research ethics and governance approval including a waiver for written informed consent from participants in this study were granted by the Western Sydney Local Health District Human Research Ethics Committee (2020/ETH02426 and 2020/ETH00786).

### Reporting summary

Further information on research design is available in the Nature Research Reporting Summary linked to this article.

## Supplementary information

Supplementary Information

Description of Additional Supplementary Files

Supplementary Data 1

Supplementary Data 2

Supplementary Data 3

Reporting Summary

## Data Availability

The host DNA depleted FastQ files for all genomes produced in this study have been deposited in the National Center for Biotechnology Information (NCBI) GenBank (BioProject PRJNA633948). File accession details for each specimen are available in Supplementary Table 1. A complete list of SARS-CoV-2 genomes sourced from GISAID (www.gisaid.org) is available in Supplementary Data 1. Source data are provided with this paper.

## Source data

Source Data
