# Supplementary material for: Co-infection with SARS-CoV-2 Omicron and Delta variants revealed by genomic surveillance
Source: Nat Commun. 2022 May 18;13:2745. doi: 10.1038/s41467-022-30518-x (PMC9117272; doi:10.1038/s41467-022-30518-x)

Supplementary Information:

Co-infection with SARS-CoV-2 Omicron and Delta Variants Revealed by Genomic Surveillance

Rebecca J Rockett[#], Jenny Draper[#], Mailie Gall[#], Eby M Sim[#], Alicia Arnott, Jessica E Agius, Jessica Johnson-Mackinnon, Winkie Fong, Elena Martinez, Alexander P Drew, Clement Lee, Christine Ngo, Marc Ramsperger, Andrew N Ginn, Qinning Wang, Michael Fennell, Danny Ko, Linda Hueston, Lukas Kairaitis, Edward C Holmes, Matthew N O'Sullivan, Sharon C-A Chen, Jen Kok, Dominic E Dwyer, Vitali Sintchenko

Table of Contents

Supplementary Appendix, April 2022 .... Error! Bookmark not defined.
SUPPLEMENTARY RESULTS **2**
Frequency of SARS-CoV-2 variant heterozygosity 2
Investigation of SARS-CoV-2 amplification bias in Omicron and Delta co-infection 2
Investigation of within-host recombination events 3
SUPPLEMENTARY REFERENCES **3**
Supplementary Table 1. 5
Supplementary Data 1. .... **Error! Bookmark not defined.**
Supplementary Data 2. .... **Error! Bookmark not defined.**
Supplementary Data 3. .... **Error! Bookmark not defined.**
Supplementary Figure 1. 6
Supplementary Figure 2. 7
Supplementary Figure 3. 8
Supplementary Figure 4. 9

SUPPLEMENTARY RESULTS

Frequency of SARS-CoV-2 variant heterozygosity

From a total of 22,942 high quality genomes (minimum 90% coverage of the SARS-CoV-2 genome) sequenced between 25 February 2020 and 20 January 2022, only 10 (0.04%) had 30 or more heterozygous sites (min 0, median 1, mean 1.28, max 77, 80th percentile 2, 99th percentile 6, 99.9th percentile 17) (Supplementary Figure 2). Sequences with ≥ 10 heterozygous sites trigger a sequence quality review. A total of 33 heterozygous sites were initially detected in the genome of Case A (Day 2) and 46 heterozygous sites from Case B (Day 3). Failure to pass this bioinformatic quality metric resulted in a complete repeat of the SARS-CoV-2 genomic workflow for these samples, including re-extraction of the original primary specimen.

Investigation of SARS-CoV-2 amplification bias in Omicron and Delta co-infection

Amplification biases noted in ONT and Illumina data are caused by lineage specific mutations impacting the efficiency of amplification primers. These biases resulted in increased variation in the frequency of lineage defining mutations, particularly when comparing amplification-based SARS-CoV-2 enrichment to probe capture-based methodologies (Supplementary Figures 3 and 4). Amplification of Omicron subpopulations is reduced in the region

encompassing Midnight amplicon 28 (nt 27808 – 28985). This is due to a mismatch at the 3'end of forward primer 28 (TTTGTGCTTTTTAGCCTTTCTG**C**(t-Omicron)T. Mutations in the last 5bp of primer sequences significantly impact the efficiency of amplification, therefore Delta populations were preferentially amplified over lineage markers ORF7a:V82A, ORF7a:T120I (Supplementary Table 1). Similar biases were noted in Omicron lineage markers (S:N856K, S:L981F) due to a primer mismatch in forward primer 24 (GCTGAA**C**(t-Omicron)ATGTCAACAACTC). Although not a lineage defining mutation, a 17-bp deletion in ORF7a predominated in Delta strains circulating in Australia between July and December 2021. This deletion was not detected in the Delta-predominant consensus genomes of Case B, Day 11 in genomes produced using Midnight primer amplification, but was present in the genomes produced using RVOP. A Delta-specific mutation in the Midnight reverse primer 27 at the 3'end of the primer was detected, likely causing preferential amplification of the Omicron subpopulation in this region, encompassing Midnight amplicon 27. A similar phenomenon was recently reported for a collection of Delta samples with minor Omicron contamination sequenced using ARTIC V3.[20]

Investigation of within-host recombination events

To further investigate any possible recombination events, we performed a number of additional bioinformatic analysis. We identified 8 sites that encode mutations within the span of a single 75bp read, to uncover if mutations in a single read encoded both Omicron and Delta defining mutations. This analysis was performed on deduplicated RVOP reference aligned reads (Supplementary Data 3). We also confirmed the lack of recombination signatures by read based phasing using WhatsHap[1] (version 1.2) within non-overlapping midnight amplicon regions (ONT and Illumina). Both approaches confirmed that at the timepoints sampled in these two infections, no recombination events could be detected.

SUPPLEMENTARY REFERENCES

1. Martin, M. *et al.* WhatsHap: fast and accurate read-based phasing. *bioRxiv* 085050 (2016) doi:10.1101/085050.

SUPPLEMENTARY TABLES, DATA AND FIGURE LEGENDS

Supplementary Table 1. SARS-CoV-2 yield and sequencing metrics for co-infection cases

SRA Accession	Case ID	DOC	Day of infection	Ct	log10 viral load	Sample type	Method	Covered bases	Average Depth	Culture result	SARS-CoV-2 amplification method	Pango (probability)
SRR18006919	A	12/31/21	2	17.33	8.0	Nasopharyngeal swab	illumina	29395	9962x	Not performed	Midnight	AY.101
SRR18006922	A	01/01/22	3	23.44	5.6	Nasopharyngeal swab	illumina	29401	11171x	Positive	Midnight	None
SRR18006916	B	01/03/22	3	19.26	7.2	Nasopharyngeal swab	illumina	29401	11152x	No growth	Midnight	None
SRR18006913	B	01/11/22	11	24.05	5.4	Nasopharyngeal swab	illumina	29329	8244x	Not performed	Midnight	AY.39.1
SRR18006925	A	12/31/21	2	17.33	8.0	Nasopharyngeal swab	ONT	29714	336.76x	Not performed	Midnight	AY.35(0.99)
SRR18006912	A	01/01/22	3	23.44	5.6	Nasopharyngeal swab	ONT	29714	305.82x	Positive	Midnight	None
SRR18006918	B	01/03/22	3	19.26	7.2	Nasopharyngeal swab	ONT	29714	328.788x	No growth	Midnight	None
SRR18006915	B	01/11/22	11	24.05	5.4	Nasopharyngeal swab	ONT	29713	272.737x	Not performed	Midnight	AY.39.1 (0.97)
SRR18006924	A	12/31/21	2	17.33	8.0	Nasopharyngeal swab	RVOP	29901	590x	Not performed	hybrid-capture	B.1 (0.86)
SRR18006923	A	01/01/22	3	23.44	5.6	Nasopharyngeal swab	RVOP	29901	4899x	Positive	hybrid-capture	B.1 (0.45)
SRR18006917	B	01/03/22	3	19.26	7.2	Nasopharyngeal swab	RVOP	29901	2000x	No growth	hybrid-capture	B.1 (0.99)
SRR18006914	B	01/11/22	11	24.05	5.4	Nasopharyngeal swab	RVOP	29900	142x	Not performed	hybrid-capture	B.1.95 (0.16)
SRR18006920	A	01/01/22	3	15.37	8.8	Cultured isolate	illumina	28685	9754x	Positive	Midnight	AY.39.1
SRR18006921	B	12/31/21	0	31.68	2.4	Nasopharyngeal swab	illumina	26669	7086x	Not performed	Midnight	B.1.1.529

Supplementary Figure 1. Log-scale histogram of heterozygosity in 22,942 high quality (≥90% SARS-CoV-2 genome coverage) SARS-CoV-2 genomes produced from NSW, Australia. Black dots represent the number genomes containing heterozygous bases (0 – 80 sites per genome). Larger dark blue dots indicate the genomes investigated in this study, the regression is depicted by the blue line, with the grey area showing the 95% confidence intervals, the red line indicates our quality control threshold where genomes undergo case review if >10 heterozygous bases are detected.

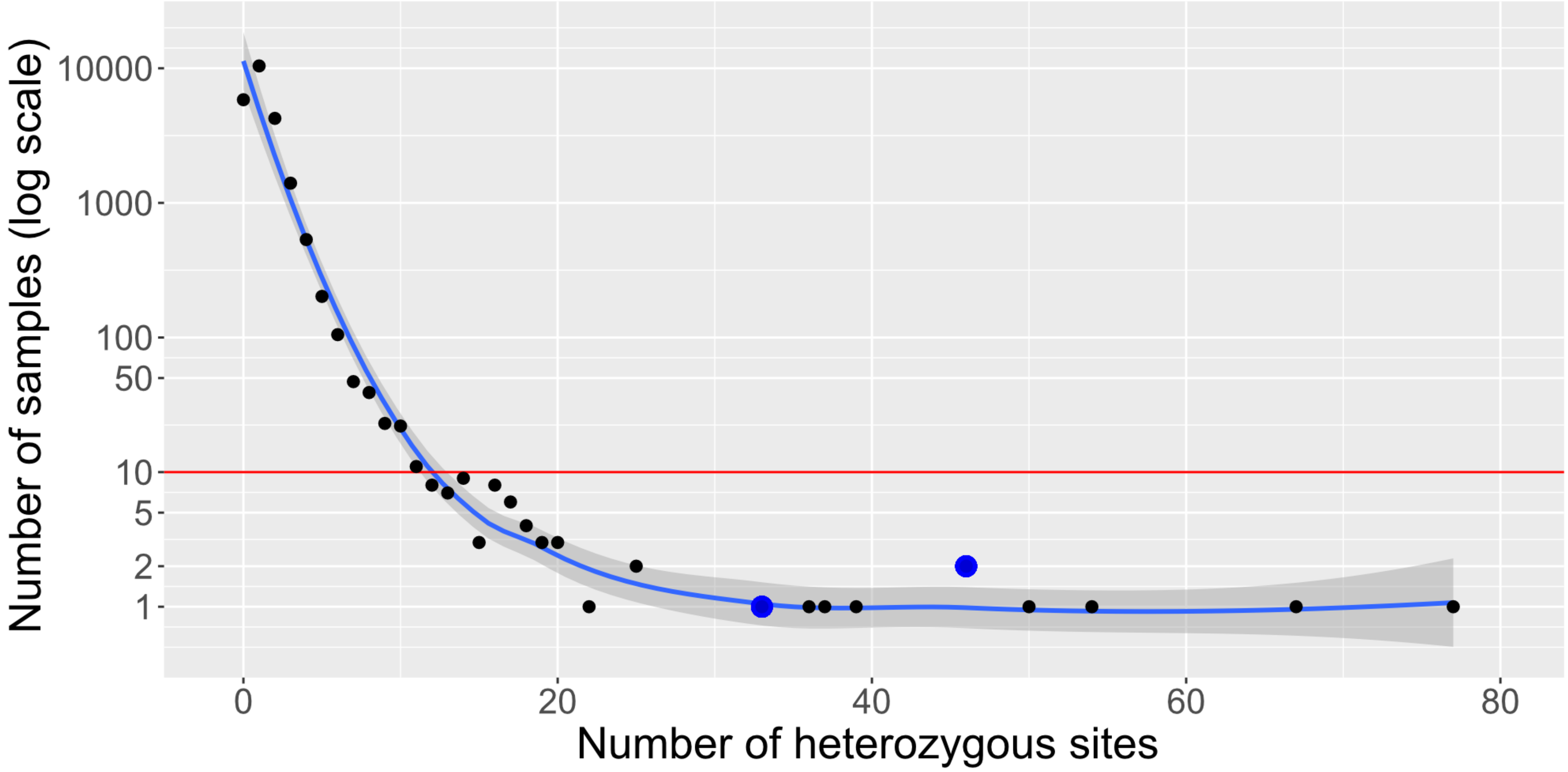

Supplementary Figure 2. Comparison of sensitivity of different sequencing methods and technologies to detect SARS-CoV-2 co-infection with the Delta and Omicron lineages. Average population frequency of the Omicron (yellow) and Delta-lineage (blue) defining mutations in clinical samples by different sequencing method. Segments in grey represent differences in Omicron and Delta mutational frequencies.

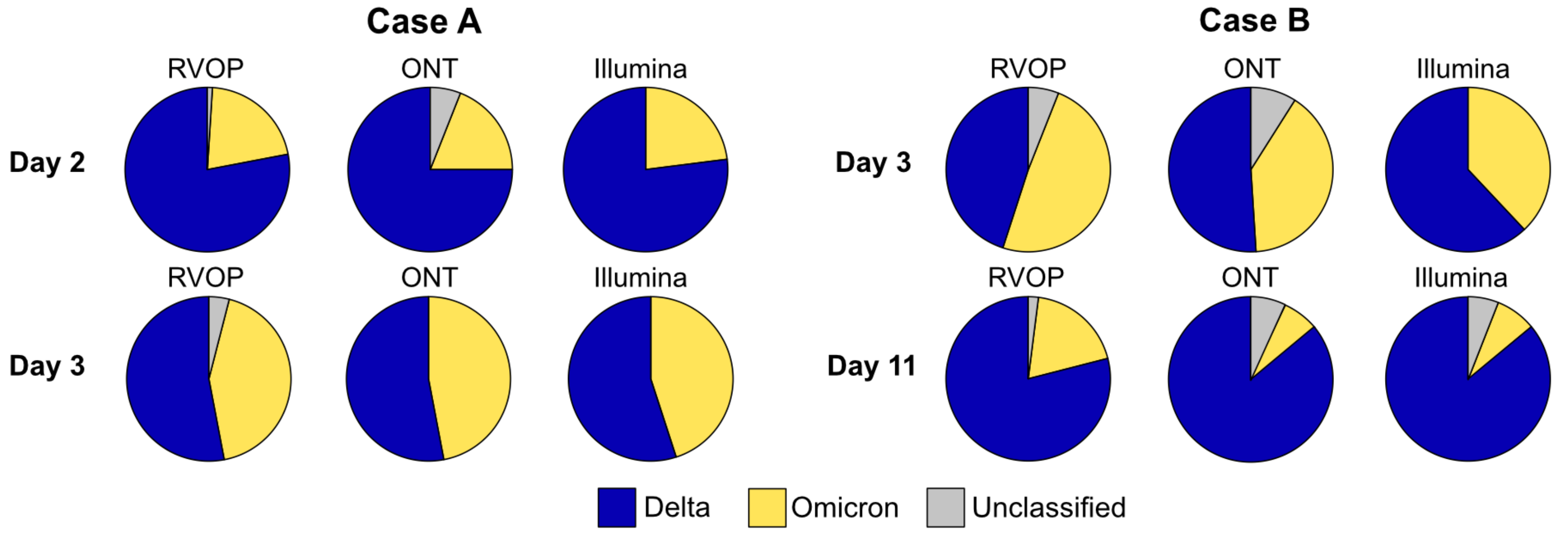

Supplementary Figure 3. Read frequency distribution of Delta and Omicron lineage markers using three SARS-CoV-2 sequencing methods. Both Delta (blue) and Omicron (yellow) lineages markers were detected **Case A** on Day 2, with Delta being the dominant lineage (P=0.00265 (Illumina), P=0.00267 (ONT), P=0.000003 (RVOP)). On Day 3, the difference was reduced, with no difference in the proportion of Delta / Omicron detected by Illumina (P=0.267) or ONT (P=0.941). RVOP still detected a significant difference in the proportion of Delta and Omicron lineage markers (P=0.0002). For **Case B** on Day 3 - there was more Delta than Omicron lineage detected by Illumina sequencing (P=0.0167), but no difference in the proportion of Delta/Omicron detected by ONT (P=0.431) or RVOP (P=0.169). By Day 11 the proportion of Delta had increased as detected by all three-sequencing methods (P=0.0004 (Illumina), P=0.0006 (ONT), $P=1.3 \times 10^{-12}$ (RVOP)). For each specimen, allele frequencies derived from different sequencing methods were compiled from 10 and 17 mutations unique to Delta and Omicron respectively. Median frequencies are represented by bold black lines within each box, the box limits indicate the 25th and 75th percentiles and the whiskers extend to the minimum and maximum allele frequencies. Statistical analysis was performed by Welch Two Sample t-test, without multiple testing correction. NS (not significant) $p > 0.05$, * $p \leq 0.05$, *** $p \leq 0.001$. Sequencing methods: ONT - Oxford Nanopore Technologies sequencing, Illumina – Illumina short read sequencing, RVOP - Respiratory Virus Oligo Panel.

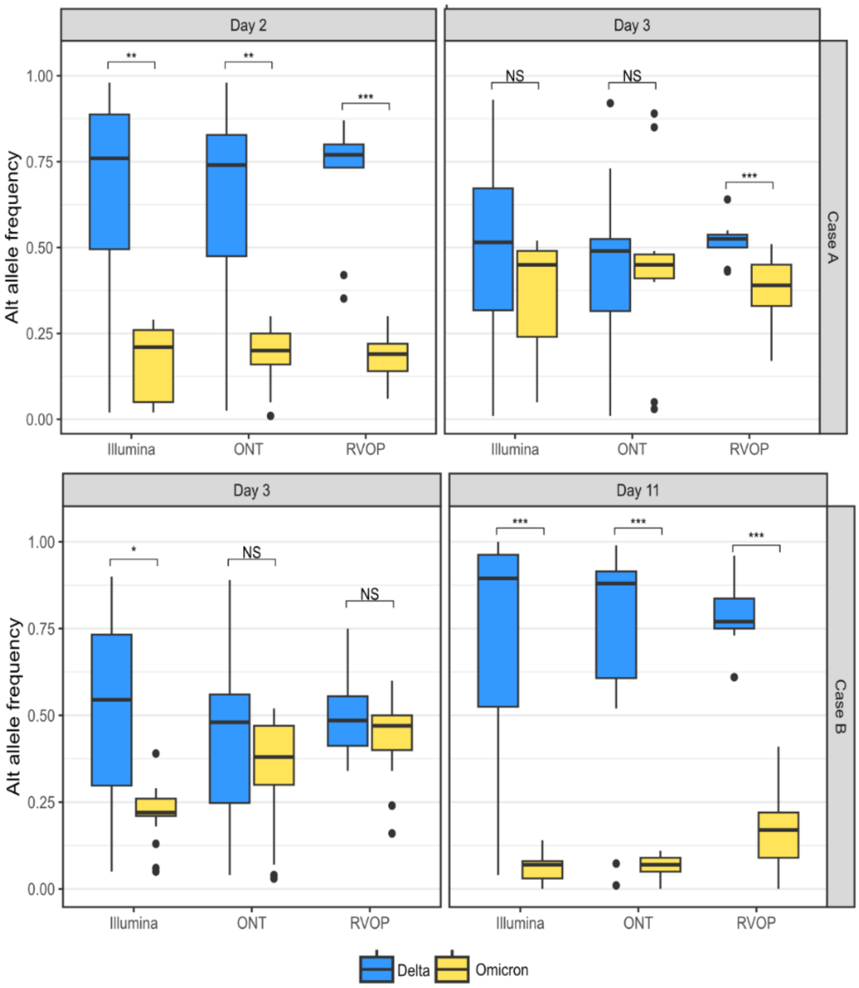

Supplementary Figure 4. Genome-wide view of SARS-CoV-2 with the variant frequency of Delta and Omicron lineage-defining polymorphisms in specimens sequenced using three SARS-CoV-2 sequencing methodologies. A total of 27 polymorphism defining Delta and Omicron lineages are shown in relation to the annotated SARS-CoV-2 genome. The frequency of sequencing reads encoding each mutation is shown by histograms highlighting the constellation of mutations defining each lineage. Blue bars demonstrate the frequency of mutations defining the Delta lineage and yellow bars the Omicron lineage. Due to the close genomic location of lineage defining mutations in the spike region some bars are overlapping. Read frequencies are collected from ONT, Illumina and RVOP data and although there is high concordance between sequencing methods and technologies, the dashed line inserts indicate regions of amplification bias that preferentially amplify Delta or Omicron in ONT and Illumina sequencing results, in contrast to RVOP data where lineage defining mutational frequencies remain more consistent.

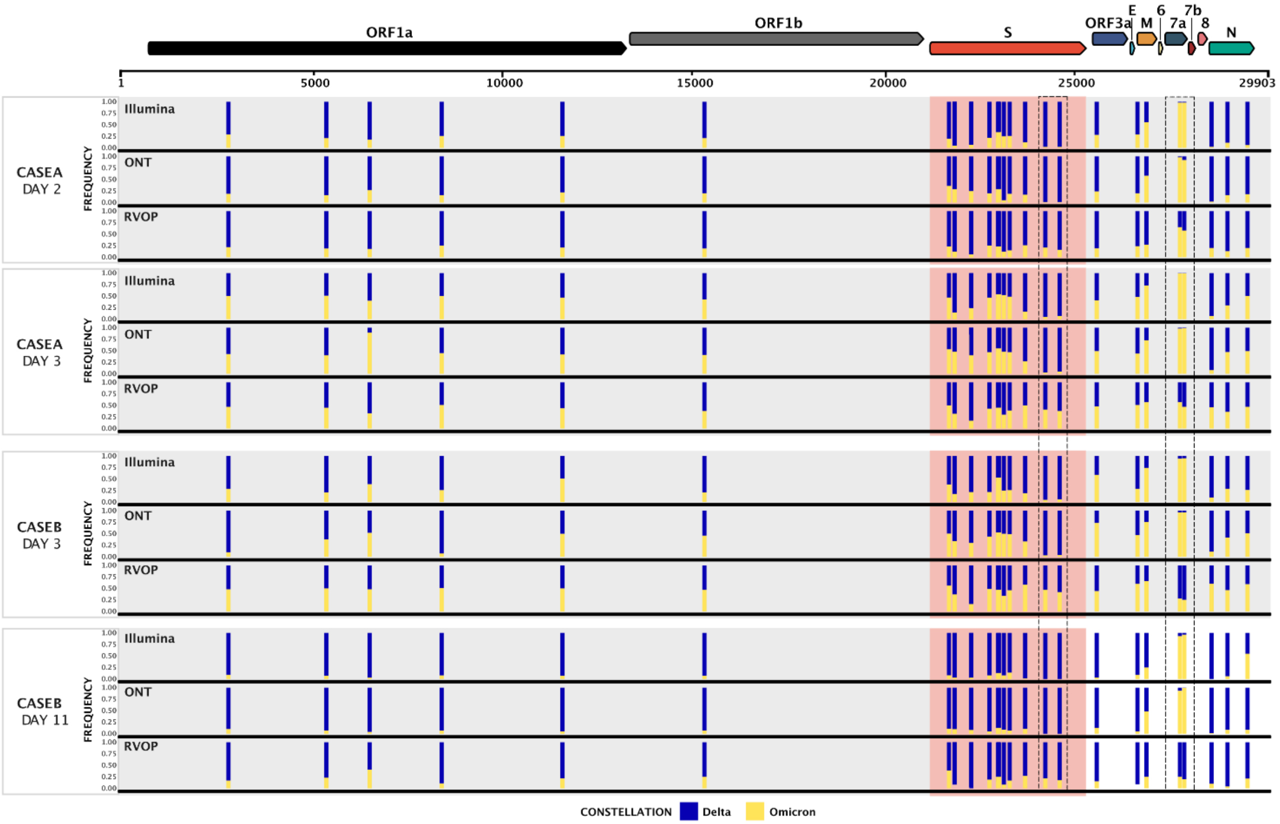

Supplement: Supplementary file 1 — Supplementary Information [file 41467_2022_30518_MOESM1_ESM.pdf]
