# Supplementary material for: Co-infection with SARS-CoV-2 Omicron and Delta variants revealed by genomic surveillance
Source: Nat Commun. 2022 May 18;13:2745. doi: 10.1038/s41467-022-30518-x (PMC9117272; doi:10.1038/s41467-022-30518-x)
Supplement: Supplementary file 2 — Description of Additional Supplementary Files [file 41467_2022_30518_MOESM2_ESM.docx]

**Supplementary data legends**

**Supplementary Data 1.** Acknowledgement of SARS-CoV-2 genomes used in the study, generously contributed by international laboratories on the GISAID repository**.**

Provided in Supplementary Data 1

**Supplementary Data 2.** List of coordinates and amino acid changes supported by forward and reversed reads based on RVOP data.

Provided in Supplementary Data 2

**Supplementary Data 3.** List of mutations coded within a single read to investigate within host recombination.

Provided in Supplementary Data 3.
