# Supplementary material for: Co-infection with SARS-CoV-2 Omicron and Delta variants revealed by genomic surveillance
Source: Nat Commun. 2022 May 18;13:2745. doi: 10.1038/s41467-022-30518-x (PMC9117272; doi:10.1038/s41467-022-30518-x)
Supplement: Supplementary file 6 — Reporting Summary [file 41467_2022_30518_MOESM6_ESM.pdf]

nature research

Corresponding author(s): Vitali Sintchenko
Last updated by author(s): April 14, 2022

Reporting Summary

Nature Research wishes to improve the reproducibility of the work that we publish. This form provides structure for consistency and transparency in reporting. For further information on Nature Research policies, see Authors & Referees and the Editorial Policy Checklist .

Statistics

For all statistical analyses, confirm that the following items are present in the figure legend, table legend, main text, or Methods section.

n/a	Confirmed	
☐	☒	The exact sample size (*n*) for each experimental group/condition, given as a discrete number and unit of measurement
☐	☒	A statement on whether measurements were taken from distinct samples or whether the same sample was measured repeatedly
☐	☒	The statistical test(s) used AND whether they are one- or two-sided<br>*Only common tests should be described solely by name; describe more complex techniques in the Methods section.*
☒	☐	A description of all covariates tested
☒	☐	A description of any assumptions or corrections, such as tests of normality and adjustment for multiple comparisons
☐	☒	A full description of the statistical parameters including central tendency (e.g. means) or other basic estimates (e.g. regression coefficient) AND variation (e.g. standard deviation) or associated estimates of uncertainty (e.g. confidence intervals)
☐	☒	For null hypothesis testing, the test statistic (e.g. *F*, *t*, *r*) with confidence intervals, effect sizes, degrees of freedom and *P* value noted<br>*Give P values as exact values whenever suitable.*
☒	☐	For Bayesian analysis, information on the choice of priors and Markov chain Monte Carlo settings
☒	☐	For hierarchical and complex designs, identification of the appropriate level for tests and full reporting of outcomes
☒	☐	Estimates of effect sizes (e.g. Cohen's *d*, Pearson's *r*), indicating how they were calculated

Our web collection on statistics for biologists contains articles on many of the points above.

Software and code

Policy information about availability of computer code

Data collection	No software was used for data collection, 1076 near complete global genomes were sourced from GISAID with collection dates between December 2020 and 31st December 2022 (www.gisaid.org). A complete list of GISAID genomes used is available in Supplementary Table S2
Data analysis	Raw sequence data were processed as follows: quality trimmed using Trimmomatic v0.36 (sliding window of 4, minimum read quality score of 20, leading/trailing quality of 5 and minimum length of 36 after trimming). Reads were mapped to the reference SARS-CoV-2 genome (NCBI GenBank accession MN908947.3) using Burrows-Wheeler Aligner (BWA)-mem version 0.7.17, with unmapped reads discarded. Variant calling and the generation of consensus sequences was conducted using iVar8, with soft clipping over primer regions (version 1.2.1, min. read depth >10x, quality >20, min frequency threshold of 0.1). Host reads were removed from FASTQ files before being uploaded to SRA. SARS-CoV-2 lineages were inferred using Phylogenetic Assignment of Named Global Outbreak LINeages v1.2.86 (PANGO and PLEARN). ONT bioinformatic quality control and consensus sequence were generated post run using the wf-artic workflow version 0.3.9. Mapping was visualised on the Integrative Genomics Viewer12 version 2.8.6 and ONT data was parsed using bam-readcount version 1.0. SARS-CoV-2 diversity was conducted using a global subsampling strategy developed by Nextstrain(EpiCoV). Consensus genomes were aligned with MAFFT v7.402 (FFT-NS-2, progressive method) and phylogenetic analysis was performed using the maximum likelihood approach (IQTree v1.6.7 (substitution model: GTR+F+R2) with 1,000 bootstrap replicates. The phylogenetic tree was visualised using the R package ggtree. Statistical analysis of the read distribution of Delta and Omicron lineage markers was performed by Student's t-Test using R software version 4.1.2. Graphs were generated using the package 'ggplot 2' version 3.3.5. ONT Sequencing was performed on the GridION platform running MinKNOW version 21.05.25 with live base-calling on high accuracy mode with demultiplexing enabled (Guppy version 5.0.16). Sequencing run status was monitored on board MinKNOW and run was terminated after more than 20 MB of passed base-called data was obtained per sample. Quality control and consensus sequence were generated post run using the wf-artic workflow version 0.3.9 (https://github.com/epi2me-labs/wf-artic). To determine and quantify positional heterozygosity, mapping files generated by the wf-artic workflow were visualised on the Integrative Genomics Viewer25 version 2.8.6 and parsed using bam-readcount version 1.0.1 (https://github.com/genome/bam-readcount). The phylogenetic tree was visualised using the R package ggtree version 1.99.1.

For manuscripts utilizing custom algorithms or software that are central to the research but not yet described in published literature, software must be made available to editors/reviewers. We strongly encourage code deposition in a community repository (e.g. GitHub). See the Nature Research guidelines for submitting code & software for further information.

Data

Policy information about availability of data

All manuscripts must include a data availability statement. This statement should provide the following information, where applicable:

- Accession codes, unique identifiers, or web links for publicly available datasets
- A list of figures that have associated raw data
- A description of any restrictions on data availability

Data availability statement was added to the main text.

Field-specific reporting

Please select the one below that is the best fit for your research. If you are not sure, read the appropriate sections before making your selection.

☒ Life sciences ☐ Behavioural & social sciences ☐ Ecological, evolutionary & environmental sciences

For a reference copy of the document with all sections, see nature.com/documents/nr-reporting-summary-flat.pdf

Life sciences study design

All studies must disclose on these points even when the disclosure is negative.

Sample size	This was an observational study and therefore sample size calculations were not conducted.
Data exclusions	Specimens with insufficient SARS-CoV-2 viral load to generate high quality SARS-CoV-2 genomes were excluded from the analysis.
Replication	Additional specimens (n=2, total per case n=3) were sourced to confirm the initial co-infection with VOCs Delta and Omicron
Randomization	This was an observational study not an intervention study therefore randomization was not required.
Blinding	Scientists involved in genome data analysis were not blinded to the epidemiological data in order to establish initial criteria for viral genome clustering

Reporting for specific materials, systems and methods

We require information from authors about some types of materials, experimental systems and methods used in many studies. Here, indicate whether each material, system or method listed is relevant to your study. If you are not sure if a list item applies to your research, read the appropriate section before selecting a response.

Materials & experimental systems

n/a	Involved in the study
☒	☐ Antibodies
☐	☒ Eukaryotic cell lines
☒	☐ Palaeontology
☒	☐ Animals and other organisms
☐	☒ Human research participants
☒	☐ Clinical data

Methods

n/a	Involved in the study
☒	☐ ChIP-seq
☒	☐ Flow cytometry
☒	☐ MRI-based neuroimaging

We observed Vero E6 cells are used in the study. Please check the box "involved in the study" and provide the necessary information in the appropriate module.

Ethical and governance approval including a wavier for written informed consent from participants in this study was granted by the Western Sydney Local Health District Human Research Ethics Committee (2020/ETH02426 and 2020/ETH00786).
